# Clinical observation of Long chiropractic treatment on patients with neurogenic cervical spondylosis: Study protocol for a randomized controlled trial

**DOI:** 10.1097/MD.0000000000028861

**Published:** 2022-03-04

**Authors:** Chen Wang, Zhongping Gu, Junwu Yu, Peizhen Zhang, Faming Yang

**Affiliations:** a School of Sport Medicine and Rehabilitation, Beijing Sport University, Beijing, China; b Department of Rehabilitation, Faculty of Health Services and Management, Ningbo College of Health Science, Ningbo, China.

**Keywords:** Long manipulation, muscle fatigue, neurogenic cervical spondylosis, pain, sling exercise training

## Abstract

Neurogenic cervical spondylosis is the most common type of cervical spondylosis, accounting for approximately 60% percent of the incidence of cervical spondylosis. Cervical spine Long manipulation and sling exercise training (SET) have obtained good therapeutic results in clinical rehabilitation. The aim of this study was to evaluate the effect of Long manipulation combined with SET on neurogenic cervical spondylosis. In this assessor-blind, randomized controlled trial, 90 eligible patients will be randomized into a combination treatment group (Long manipulation combined with SET), a Long manipulation group and a conventional massage group. The visual analogue score, the Neck Disability Index score, and muscle fatigue in the bilateral upper oblique and Musculus sternocleidomastoideus, using mean power frequency and median frequency from the surface electromyography frequency domain index, will be assessed before and after the intervention at 0 and 4 weeks, respectively.

**Trial registration:** Registered in the Chinese Clinical Trial Registration Center with the number ChiCTR2100054978. Registered December 30, 2021.

## 1 Introduction

Research has confirmed that the degree of muscle fatigue is also associated to its stiffness, which is a manifestation of muscle tension and increases as muscle fatigue increases. The onset of cervical spondylosis is the result of a series of symptoms and signs caused by an imbalance between the cervical vertebrae and the adjacent soft tissues, and is the result of muscle fatigue caused by long-term tension in the neck and shoulder muscles.^[1]^ Due to degenerative changes in the cervical intervertebral discs, growth of the hook or synovial joints, and hypertrophic bone spurs protruding laterally, the nerve roots at the corresponding level are stimulated or compressed, and a series of diagnostic manifestations of nerve root irritation or dysfunction of the corresponding segment occur, characterized by pain in the back of the shoulder, radiating pain in the upper limbs and fingers, numbness and weakness, etc, which are more common unilaterally, and the prognosis is mostly good, but these clinical symptoms seriously affect people's quality of life.^[2]^

At present, there are many nondrug conservative treatment methods, such as acupuncture, massage, herbal compresses, low frequency therapy, and microwaves, all of which are effective. Numerous studies have shown that the clinical efficacy of conservative treatment and surgical treatment is the identical.^[3]^ Therefore, it is important to investigate how best combined conservative treatment can be used to improve neck pain and nerve root symptoms in patients with radiculopathy and to alleviate the progression of cervical spondylosis.

Sling exercise training (SET) stresses the use of the patient's own gravity to adjust the overall biomechanics.^[1]^ It is a closed chain exercise, based on neuromuscular stimulation, to adjust the central nervous system's control of the muscles and regain function to the inactivated local stabilizing muscles. It is also a painless form of closed chain training in both “hold” and “repetition” to stimulate the deeper stabilizing muscles of the cervical spine, ultimately re-establishing the appropriate muscle control pattern to enhance the stability of the cervical spine, so that the weak chains of the muscles that make up the “upper cross” are strengthened and their compensation is relieved. The treatment therefore trains the weak chain of muscles, while improving local pain and stiffness, and thus relieving fatigue.^[1]^ There are some studies have shown that osteopathic manipulation can adjust the static balance of spinal, while tendon manipulation can adjust the dynamic balance of the neck.^[4]^ Long manipulation combines osteopathic manipulation and tendon manipulation to bring the local imbalanced bio-mechanical equilibrium into balance, release the nerve roots and their surrounding soft tissue adhesion, change the area of the intervertebral space, adjust the relationship between the hook vertebral joints, link muscle spasm, release synovial inclusions, adjust the pressure distribution of the cervical spine tissues, enhance the stability of the cervical spine, and restore the normal physiological curvature of the cervical spine.^[5]^ Therefore, this study will use SET combined with Long manipulation to observe its effect on improving pain, cervical spine function, and muscle fatigue in patients with neurogenic cervical spondylosis (NCS).

## 2 Method/design

**Figure 1:**
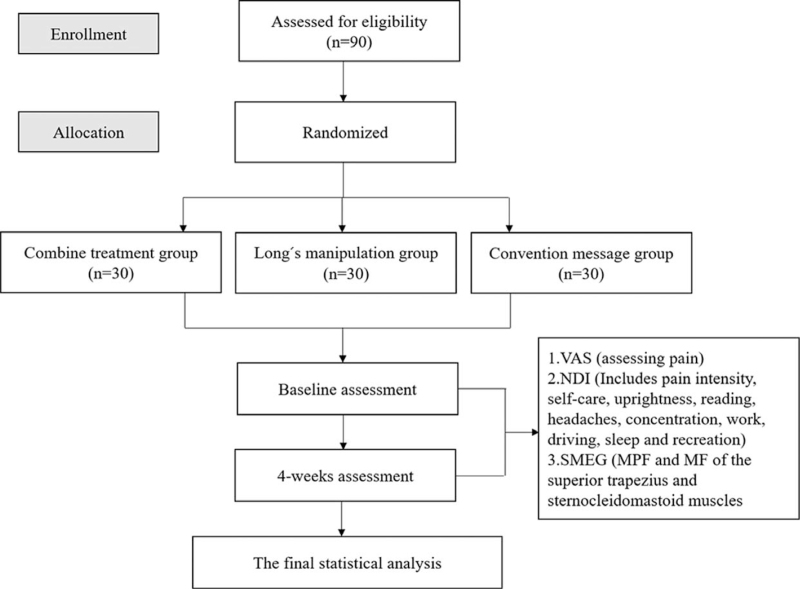
Consort diagram flowchart. MF = median frequency, MPF = mean power frequency, NDI = Neck Disability Index, SMEG = surface electromyography, VAS = visual analogue score.

The study was an assessor-blind, single-center randomized controlled trial. The aim of this study is to investigate the effect of SET combined with Long manipulation on pain, cervical function and muscle fatigue in patients with CRS. The project leader will be responsible for the overall project and will initiate the establishment of the subject project team. The subject project team will be responsible for subject security, recruitment and treatment and assessment, and quality control. The coordinating center staff will be responsible for communication of protocol changes and provision of information. The trial will have a 4-week period. After randomization, subjects will conduct assessments of primary and secondary indicators in 2 phases at 0 and 4 weeks (Fig. 1; Additional File 1).

### 2.1 Eligibility criteria

#### 2.1.1 Inclusion criteria

1. The selected patients must meet the diagnostic criteria for NCS as revised in the Second National Symposium on Cervical Spondylosis in 1993 and the modified criteria in the minutes of the Third National Symposium on Cervical Spondylosis in 2008^[6]^;
2. Having more typical ridiculous symptoms such as pain and numbness in the arm, the extent of which corresponds to the area innervated by the cervical spinal nerve;
3. The patient has functional limitation of neck movement and pressure pain in the spine process of the dysfunction vertebrae, para-vertebral area, and the internal superior scapular angle of the affected side;
4. A positive cervical compression test and/or brachial plexus nerve pull test;
5. The imaging findings are consistent with diagnosis presentations.

#### 2.1.2 Exclusion criteria

1. Patients with serious chemical diseases.
2. Patients with severe osteoporosis, anthropolising spondylitis, neoplasms, fractures and other pathologies.
3. Those who do not collaborate with study.

Any potential subjects who meet the inclusion and exclusion criteria will be recruited from the outpatient and inpatient departments of the Department of Rehabilitation Medicine at Ningbo Haishu Rehabilitation Hospital. Potential subjects who meet the requirements will be interviewed by the research staff and informed of the inclusion criteria and procedures. Eligible subjects will first be analyzed through a baseline assessment and then diagnosed based on clinical presentation and physical examination, combined with imaging studies. Subjects will be informed that participation in the trial is strictly voluntary and that they can quit from the trial at any time. If you withdraw, the data collected will not be removed and will be used for the final statistical analysis. A summary table of data containing all variables and potential risks will be created by the research center. The raw information obtained will be stored in an electronic database for subsequent statistical analysis. Subject recruitment for the study will begin on December 31, 2021. A flow chart of the trial for subjects is shown in Figure 1.

#### 2.1.3 Ethics approval and consent to participate

The study will be conducted in strict accordance with the principles of the Declaration of Helsinki on Clinical Research.^[7]^ The trial protocol has been endorsed by the Research Ethics Committee of Ningbo College of Health Science (2021-12-07). All participants will be given sufficient time to sign an informed consent form prior to studying.^[8,9]^ This study protocol has been registered with the Chinese Clinical Trials Registry (ClinicalTrials.gov, ID: ChiCTR2100054978).

#### 2.1.4 Intervention

The control group will receive conventional message treatment: the treatment plan is based on “Tui-na”, edited by Juntao Yan^[10]^: the patient is seated and relaxes the muscles of the neck, shoulders, and upper back; the patient then takes and rubs the cervical tension and spasm, presses and rubs the painful points of the neck nodes with the finger, continues 3 to 5 times, then presses the acupoints of Tianzhu, Fengchi, Fengfu, cervical spine and Dazhi with the thumb, each point for 1 minute; gently presses the neck and shoulder muscles with palm rubbing, and then applies the cervical oblique pulling method and stretches at the end position for 5 seconds. The treatment is repeated 3 to 5 times. The treatment was repeated 3 to 5 times; 30 min/time, 1 time/d, 5 times/wk, 4 weeks in total.

The Long manipulation group will be treated with Long manipulation^[11]^: a 4-step intervention using the Long manipulation method of orthopedics massage: relaxation technique, orthopedics technique, strengthening technique and pain zone technique. After relaxing the cervical tissues with kneading in the recumbent position, the cervical spine will be orthopedic stabilized corresponding to the type of misalignment: cervical axis alteration and anterior-posterior slippage misalignment with lateral pushing, left-right rotation misalignment with low head shaking, lateral bending and side-swinging misalignment with lateral moving, spine gap stenosis or mixed misalignment with traction chair orthopedics correction, followed by tendon division and tendon management in the soft tissues of the neck to regain the dynamic and static balance of the cervical spine. Finally, the hurting areas of the shoulders, back and upper limbs are treated with kneading and kneading techniques. The above treatments are given for 20 minutes each time, once a day for 4 weeks.

The combined treatment group combines SET on the basis of the Long manipulation group, in which the procedure for Long manipulation is the same as above and the treatment for SET is shown as follow^[12]^: before the start of the treatment look for weak chains, determine the stability and muscle tension of the neck muscle groups and assess the muscle strength of the flexor muscle groups on both sides of the collar to develop a training protocol. Active exercise: the patient is placed in a supine position with slightly flexed knees, the thoracic spine and pelvis are immobilized with elastic cords and the head is secured with a central parting band. The patient is asked to flex the left side, flex the right side, flex the front, expand the back and rotate the neck to the maximum degree and hold for 5 seconds; each dimension is done 5 times and counted as 1 set, a total of 5 sets are completed with 30 seconds rest between sets. Slow, controlled, stable, and pain-free movements of 30 minutes, 5 times/wk for 4 weeks; The study protocol was designed in accordance with the recommendations of the standard program project: intervention trials recommendations 2013 statement. (Table 1; Additional File 2)

**Table 1:** Standard protocol items: recommendations for intervention trials (SPIRIT) figure of the Long manipulation study protocol.

	Time	
Contents	0 wk	4 wk
Screening participants	×	
Sign informed consent	×	
Randomized	×	
Interventions		
Combine treatment group	×	×
Long manipulation group	×	×
Control group	×	×
Evaluation of therapeutic effect:		
VAS score	×	×
NDI score	×	×
SMEG assessment:		
MPF and PF of the bilateral superior	×	×
Oblique and sternocleidomastoid muscles	×	×

MPF = mean power frequency, NDI = Neck Disability Index, SMEG = surface electromyography, VAS = visual analogue score.

#### 2.1.5 Randomization, allocation, and blinding

In this study, randomization was carried out using a table of random values compiled by SPSS 23.0 software, and the patients were randomly divided into 3 groups with a 1:1:1 allocation ratio: the combined treatment group (30 cases), the Long manipulation group (30 cases) and the control group (30 cases). Only the principal investigator in this study will be aware of the order of random assignment. The assessor will be a trained external rehabilitation therapist. The assessor and the patient will not be able to communicate about the intervention during the assessment. In this study, only the assessors were blinded and therefore the study did not involve the process of unblinding.

#### 2.1.6 Outcome measurement

The visual analogue score is done by drawing a straight line on a piece of white paper, with one end representing a score of 0 and 1 end representing a score of 10, and allowing the patient to choose a score based on their subjective feelings. Zero means the patient is not in any pain; below 3, indicates moderate pain; 4 to 6, more significant pain; and 7 to 10, very severe and unbearable pain.^[13]^

The Neck Disability Index score is commonly used in clinical practice to evaluate the functional status of the cervical spine. The scale includes 10 areas of pain, personal care, lifting, reading, headache, concentration, work, driving, sleep and recreation. Each item is scored out of 5, with the total score ranging from 0 (no impairment) to 50 (complete paralysis), the higher the score the greater the dysfunction.^[14]^

The surface electromyography (SMEG) was used to inspect the neck muscles of the 3 groups of patients, and the room temperature was controlled at about 24°C and the air humidity was 70% to 80%. The patient's neck skin was first disinfected with 75% of alcohol and the hair was removed with sandpaper to reduce resistance. A carbon pen was used to mark the placement of electrodes on the bilateral neck surface symmetrically.^[15]^ The SMEG signals of the upper trapezius muscle during 20 seconds of maximal voluntary contraction were used and analyzed for frequency domain indices. The patient was placed in a sitting position with his shoulders relaxed and leaning down naturally. Two nonelastic band loops were adjusted to fit over the patient's relaxed shoulders without any constraint, and the patient was made to shrug his shoulders as hard as he can.^[16]^ The sternocleidomastoid electrodes are positioned as follows^[15]^: 1 cm from the midpoint of the line between the mastoid and the sternal head, 2 surface electrodes are placed symmetrically parallel to the direction of the muscle fibers. The patient is asked to lie prone on the operating bed, with the head dangling out of the bed and both heads on either side of the body, while the patient's upper body is fastened with a nylon strap. When the patient is unable to retain the neck extension position and the weight falls to the ground, the examination ends and the duration of posterior neck extensions is recorded. SMEG data from patients during maximal isometric contractions were collected using a ME6000-T8 SMEG in all 3 groups, and then the Mega Win 3.1 software was assigned to process the data. The main analysis indexes used were mean power frequency and median frequency, which are usually closely related to the functional state of the muscle (ie, the degree of fatigue).

#### 2.1.7 Assess safety

To maintain the safety of the subjects participating in this study, the project leader will supervise the data collection process. During each monitoring session subjects will be asked to remain in hospital for 30 minutes after treatment and will be asked if there are any possible signs of discomfort during the trial. Side effects following the intervention will be recorded during each treatment session.

### 2.2 Data analysis

#### 2.2.1 Sample size calculation

The study used G∗Power version 3.1 software for sample estimation and selected the F-test, which was calculated using the MANOVA model.^[17]^ Effect size = 0.30, α=0.05, power = 0.92, group size 3 and number of repeated measures of 2 were selected to generate a sample size of 81. Taking into account a possible attrition rate of 11% during the trial, the final sample size was obtained as 90 and was allocated in a ratio of 1:1:1, that is, 30 cases in each group.

#### 2.2.2 Statistical analysis

The *Kolmogorov–Smirnov* test of normality was used for all continuous variables in the 3 groups, and if *mean* *±* *SD* were used, the 3 groups had to be normally distributed. Comparisons between groups were made using *MANOVA*. Posthoc tests were undertaken using the *SNK* method and within-group comparisons were conducted using the *paired samples t test*. If the data did not conform to a normal distribution, comparisons between groups were made using a nonparametric test for paired samples, and comparisons between groups were made using a nonparametric test for multiple samples. Data is represented as *median (interquartile range)*. For discontinuous variables, examples (%) were used and the significance level was set at *P* < .05.

#### 2.2.3 Data collection and monitoring

This study is a 4-week randomized controlled trial. Subjects will be required to undergo a 4-week clinical intervention. Two independent researchers will record during the 0 and 4-week phases and discrepancies will be resolved through discussion and third-party research institutions. The Ningbo College of Health Science will be responsible for supervision and quality control.

#### 2.2.4 Quality control

Prior to the start of the study, we will provide uniform training to all relevant doctors, nurses and assessors to ensure that they understand the entire trial process. To ensure study quality, we will send 2 supervisors per month to ensure that the enrolled subjects meet the inclusion and exclusion criteria. Adequate participation in the study will be ensured throughout the study; all enrolled subjects will follow strict clinical trial procedures, complete a case report form and follow standard operating procedures. Throughout the trial intervention, investigators will keep detailed records of withdrawals, drop-outs and related reasons, and document any compliance by subjects.

## 3 Discussion

The causes of NCS are many and complex, and in the early stages of clinical practice it was thought to be associated to mechanical compression, inflammatory responses of the nerve roots or autoimmune reactions.^[18]^ As research progressed, it was found that in the physiological state, the spinal cord, nerves and blood vessels are in a delicate balance of relaxation and fixation both inside and outside the spinal canal; there is a large space not only laterally but also anterior and posterior in the spinal canal.^[19]^ However, when degeneration occurs in the cervical spine, there is an imbalance between the bony flab and bony canal (eg, vertebral canal, vertebral artery canal, nerve root canal) and tissues within the canal (eg, spinal cords, nerves, blood vessels, etc) and the soft tissues around the neck, which has become a key trigger for the development of cervical spondylosis.^[20]^

SET uses a unique suspension device that allows the patient to train the “local stabilizing muscles” and normalize the sensorimotor control of the muscles on an unstable support surface (suspension belt), and is designed to stimulate the deep stabilizing muscles of the cervical spine and re-establish correct muscle movement control patterns to enhance cervical stability in a pain-free manner.^[19]^ In the neutral supine position, the deep posterior cervical muscles are activated to maintain the stability of the cervical spine; in the supine posterior supine position, the superficial posterior occipital and posterior cervical muscles are activated, and targeted training in these 2 positions can effectively enhance the stability of the cervical spine and in both positions, targeted training can effectively improve the stability of the cervical spine and improve the fatigue tolerance of the neck muscles.^[21,22]^

The Long technique uses a 3-step localization diagnosis, analyses the site of small joint misalignment, for different types of misalignment, uses the corresponding orthopedic repositioning techniques to intervene and treat them adherence to the principles of mechanics, which are highly targeted and can not only provide a stable, accurate and light repositioning, but also relieve or reduce the impact of disc protrusion and intervertebral space changes on the nerve roots, restore the static balance of the bones and joints of the cervical spine, and avoid unnecessary side injuries.^[23]^ Among the Long techniques, the lateral lying shoulder shaking method, the lateral head shaking method, the lateral moving method, the low head shaking method, and the supine extraction and extension pushing method can quickly, or even instantly, correct the pathological condition of abnormal joint position relationship, achieving immediate and effective clinical results.^[24]^ Jiang et al^[25]^ observed the effect of Long manipulation on the elasticity of the intervertebral disc in the cervical spine bio-mechanical model and proved that rotational manipulation could reduce the creep and strain relaxation rate of the cervical intervertebral disc and adjust the viscoelasticity and stress distribution of the cervical intervertebral disc, confirming that the manipulation could improve the stability of the cervical spine. The Long technique embodies the 2 main themes of Tui Na medicine, namely “bone and flesh” and “symptoms and essence”, and is precise, light, safe, and effective.

Therefore, we planned to observe the clinical efficacy of Long manipulation combined with SET in patients with NCS. We will also investigate how the most conservative combination therapy can be used to improve the symptoms of neck pain and NCS, and explore whether it is the first choice for conservative treatment of potential NCS.

## Author contributions

Chen Wang, Zhongping Gu, and Junwu Yu contributed equally to the design of the study, the conduct of the experiments and the writing of the manuscript. All authors were involved in the design of this study and the conduct of the experiments. Faming Yang and Peizhen Zhang are co-corresponding authors on this manuscript and contributed equally to the supervision and coordination of this clinical trial. All authors in this paper read and approved the final manuscript.

**Conceptualization**: Chen Wang, Junwu Yu, Faming Yang, Peizhen Zhang.

**Data curation**: Chen Wang, Junwu Yu, Zhongping Gu, Faming Yang.

**Formal analysis**: Junwu Yu, Zhongping Gu, Faming Yang.

**Funding acquisition**: Zhongping Gu, Junwu Yu, Faming Yang.

**Investigation**: Chen Wang, Junwu Yu, Zhongping Gu, Faming Yang.

**Methodology**: Chen Wang, Faming Yang, Peizhen Zhang.

**Project administration**: Faming Yang, Peizhen Zhang.

**Resources**: Junwu Yu, Zhongping Gu, Faming Yang.

**Software**: Chen Wang, Junwu Yu.

**Supervision**: Faming Yang.

**Validation**: Junwu Yu, Zhongping Gu, Faming Yang.

**Visualization**. Junwu Yu, Zhongping Gu, Faming Yang.

**Writing – original draft**: Chen Wang, Zhongping Gu, Junwu Yu, Faming Yang, Peizhen Zhang.

**Writing – review & editing:** Chen Wang, Faming Yang.
